# Voltammetric Ion Sensing with Ionophore-Based Ion-Selective Electrodes Containing Internal Aqueous Solution, Improving Lifetime of Sensors

**DOI:** 10.3390/membranes12111048

**Published:** 2022-10-27

**Authors:** Valentina Keresten, Konstantin Mikhelson

**Affiliations:** Chemistry Institute, Saint Petersburg State University, 26 Universitetsky Prospect, 198504 Saint Petersburg, Russia

**Keywords:** voltammetry, ion-selective electrodes, ionophores, lifetime, water uptake, potentiometry, electrochemical impedance

## Abstract

The possibility of voltammetric ion sensing is demonstrated, for the first time, for ion-selective electrodes (ISEs) containing an internal aqueous solution. ISEs selective to calcium, lithium and potassium ions are used as model systems. The internal solution of the ISEs contains a chloride salt of the respective cation and a ferrocenemethanol or ferrocyanide/ferricyanide redox couple. A platinum wire is used as the internal reference electrode. It is shown, theoretically and experimentally, that the dependence of oxidation and reduction peak potentials on the sample composition obeys the Nernst law, while the peak currents virtually do not depend on the sample composition. Thus, the electrode behavior is similar to that reported by Bakker’s group for solid contact ISEs with ultra-thin membranes (200–300 nm). It is shown that the use of classical ISEs with relatively thick membranes (100–300 µm) and internal aqueous solution allows for the sensor lifetime of about one month. It is also shown that use of a suitable background electrolyte allows for improvement of the detection limits in voltammetric measurements with ISEs.

## 1. Introduction

Ion-selective electrodes (ISEs) with ionophore-based polymer membranes are well-established electrochemical sensors [1], which are widely used in various applications from clinical analysis [2,3,4] to environmental control [5,6,7,8,9], to the analysis of food products [10,11], to agriculture and to the control of hydroponic systems [12,13]. For decades, measurements with ISEs have been performed in potentiometric (zero-current) mode. Zero-current measurements with ISEs are advantageous in view of a large working range of these sensors: the concentrations (rigorously, activities) of analytes can be quantified over several orders of magnitude of their values. Due to logarithmic transduction, a constant absolute error in the EMF measurement translates into a constant relative error in concentration. The latter, however, is relatively high because the sensitivity of potentiometric measurements is limited by the Nernst factor. For instance, an absolute error in 1 mV results in a relative error of 4% for a monovalent ion and 8% for a divalent ion.

Another limitation of the traditional analysis with ISEs is the inability to distinguish between different kinds of samples with similar ionic composition. This problem is solved by the use of arrays of several sensors. Unlike measurements with individual ISEs, the use of arrays, together with treatment of the signals by means of artificial neural networks or chemometrics (electronic tongue) allows for the characterization of samples beyond their ionic composition [4,7,8,9,11,14,15]. This makes not only quantitative but also qualitative analysis with ISEs possible. So far, however, such measurements have always been performed in zero-current mode. Depending on the particular task, arrays used in potentiometric analysis contain between 5 to 30 individual ISEs. It is therefore tempting to obtain a response on several analytes from a sole sensor, so the number of ISEs in array can be decreased.

Recent developments proved that measurements with ISEs in non-zero current mode allow for a drastic improvement of their practically important characteristics [16]. Galvanostatically polarized ISEs showed lower detection limits in the nanomolar range [17,18,19,20,21,22]. A combination of a cathodic polarization of ISEs in diluted samples with an anodic polarization at high concentrations allowed a working range of over 10 orders of magnitude with Cd-ISE to be obtained [23]. Constant potential coulometry with solid contact electrodes (SC-ISEs), proposed by Bobacka et al. [24,25,26], made it possible to achieve the sensitivity at the level less than 1%, i.e., to increase it several times in comparison with potentiometry [27,28]. It is also possible to carry out chronoamperometric measurements with classical ISEs containing internal reference solution and internal reference electrode, using capacitors connected in series to the electrodes [29,30].

It was shown that SC-ISEs with a conducting polymer (CP) in the transducer layer are also suitable for voltammetric measurements [31,32,33,34,35,36]. Furthermore, voltammetric measurements with SC-ISE made multianalyte detection possible: the same ISE can be used to determine several ions [37,38,39,40,41]. This makes voltammetry with ISEs especially promising for systems such as electronic tongue.

A theory of voltammetric response of SC-ISEs was developed by Bakker’s group [37,40,41]. Voltammetric measurements with SC-ISEs involve the use of spin-coated ultrathin membranes with thickness of 200–300 nm. Due to membrane delamination and the leak of membrane components, the lifetime of such ISEs is very short, which limits their practical application. To reduce the influence of these negative factors, it was proposed to use polyurethane instead of polyvinyl chloride as a matrix [42,43,44].

It is tempting to study classically designed ISEs with internal aqueous solution and internal reference electrode in voltammetric mode as well. Classical ISEs generally demonstrate a long lifetime (several months or even years) and excellent piece-to-piece reproducibility of their potentials. Classical ISEs can be used in potentiometric, chronoamperometric, chronopotentiometric and impedance measurements. So far, voltammetric studies with such ISEs were aimed, primarily, at the fundamental research of the mechanisms of processes at membrane/solution interfaces [45,46,47]. Here we report, for the first time, on the possibility to obtain voltammetric responses, analogous to those described by Bakker’s group, using classical ISEs. The aim of this study was to improve the lifetime of the sensors. To obtain a voltammetric response, a redox process coupled with the transfer of the analyte ion across the membrane/solution boundary is necessary. In SC-ISEs, CP is used as a redox system and its oxidation/reduction causes the analyte transfer. We use a redox system (either K_3_Fe(CN)_6_ + K_4_Fe(CN)_6_ or ferrocenemethanol (FcMeOH)) as a component of the internal filling solution and platinum internal reference electrode. We show here that Ca-, Li- and K-selective ISEs constructed in such a manner demonstrate voltammetric response, with a Nernstian potential shift of the oxidation and reduction peaks, and with lifetime of at least one month.

## 2. Theoretical Considerations

A theory of voltammetric response of SC-ISEs proposed by Bakker’s group [37], essentially, is as follows. In voltammetric mode the ion, to which the electrode is selective, is transferred from the aqueous solution to the membrane or back driven by the applied potential. During the anodic scan, CP is oxidized and the ion exchanger from the membrane compensates for the resulting positive charge of the oxidized form of CP. Consequently, the analyte ion is extracted to the solution to maintain the macroscopic electroneutrality. In the backward (cathodic) scan CP is reduced and the analyte ion returns from solution back to the membrane. Unlike in classical voltammetry, the potential applied by the instrument includes the impact which drives the redox process on the CP and also the boundary potential differences at the membrane/solution interface and membrane/CP interface. This results in the following pattern, which is significantly different from classical voltammetry: a peak current does not depend on the analyte activity in the sample (because the redox system is the CP in the SC-ISE, not in solution), whereas the peak potential changes according to Nernst equation.

The design of classical ISEs with aqueous solution and internal reference electrode is significantly different from that of solid-contact ISEs. However, as shown below, one can envisage that the voltammetric response of a classical ISE filled with a solution containing IX electrolyte, a redox-active species *Ox* and *Red* and also an inert internal reference electrode (e.g., platinum) must follow the regularities analogous to those described by Bakker et al. [37] for SC-ISEs with a CP in the transducer layer.

Let us assume that the interface between platinum electrode (*Pt*) and the internal solution, as well as interfaces between the membrane and the internal and the external solution (sample) are at electrochemical equilibrium. Then the potentials at the interface between platinum and the internal solution ($E_{Pt}$) and at the interfaces between the membrane and the external and the internal solutions ($E_{M}^{ex}$ and $E_{M}^{in}$) obey the Nernst equation with S≅2.303RT/F:

(1) $$E_{Pt}=E_{Pt}{}^{0}-Slog\frac{a_{Red}}{a_{Ox}}$$

(2) $$E_{M}^{ex}=E_{M}{}^{0}+Slog\frac{a_{I}^{ex}}{a_{I}^{mem}}$$

(3) $$E_{M}^{in}=-E_{M}{}^{0}+Slog\frac{a_{I}^{mem}}{a_{I}^{in}}$$

Terms $E_{Pt}^{0}$ and $E_{M}^{0}$ are determined by the standard chemical potentials of the species involved in the formation of interfacial electrical potentials and are assumed constant. Values $a_{Red}$, $a_{Ox}$, $a_{I}^{ex}$, $a_{I}^{in}$ and $a_{I}^{mem}$ refer, respectively, to the activities of *Red* and *Ox* in the internal solution and the activity of *I^+^* in the external and internal aqueous solutions and in the membrane.

When the applied potential is scanned to more positive values the *Red* species is oxidized to *Ox*, and an electron transfers from the internal solution to platinum electrode. The respective positive charge is compensated by the transfer of *I^+^* cation from the internal solution across the membrane to the external aqueous phase. In this way, the current flows through classical ISE. For the external voltage *E_applied_*, we obtain:

(4) $$E_{applied}=Const+E_{Pt}+E_{M}^{ex}+E_{M}^{in}=Const+E_{Pt}{}^{0}+S\left(log\frac{a_{I}^{ex}}{a_{I}^{mem}}+log\frac{a_{I}^{mem}}{a_{I}^{in}}-log\frac{a_{Red}}{a_{Ox}}\right)$$

The term Const combines the contributions from the reference electrode (e.g., Ag/AgCl in 3.5 M KCl) and from the liquid junction potential at the contact of the salt bridge with the solution; these contributions are considered constant. The total concentration of redox-active species in the internal solution is constant: $C_{RedOx}{}^{tot}=C_{Red}+C_{Ox}$. The total concentration of cationic species (*I^+^* and *Ox*) in the internal solution is also constant and equals the concentration of X^−^ anions: $C_{I}^{in}+C_{Ox}=C_{X}^{in}$. Equation (4) therefore can be re-written as

(5) $$E_{applied}-Sloga_{I}^{ex}=Const+E_{Pt}{}^{0}-S\frac{\left(\left(C_{RedOx}{}^{tot}-C_{Ox}\right)/\gamma_{Red}^{in}\right)\left(\left(C_{X}{}^{in}-C_{Ox}\right)/\gamma_{I}^{in}\right)}{a_{Ox}}$$

Symbol γ stands for the respective activity coefficients. This equation is fully equivalent to Equation (6) in [37] and therefore the regularities in the behavior of classical ISEs in voltammetric mode of measurements must be the same as shown earlier for SC-ISEs with CPs in the transducer layer.

## 3. Materials and Methods

Potassium ionophore I, valinomycin; calcium ionophore I, diethyl N,N′-[(4R,5R)-4,5-dimethyl-1,8-dioxo-3,6-dioxaoctamethylene]bis(12-methylaminododecanoate)] (ETH 1001); lithium ionophore VIII, 2-[2,2-bis[[2-(dicyclohexylamino)-2-oxoethoxy]methyl]butoxy]-N,N-dicyclohexylacetamide; cation exchangers tetrakis-p-chlorophenylborate (KClTPB) and sodium tetrakis-[3,5-bis(trifluoromethyl)phenyl]borate (NaTFPB), tetradodecylammonium tetrakis(4-chlorophenyl)borate (ETH 500), plasticizers bis(2-ethylhexyl)sebacate (DOS), 2-nitrophenyloctylether (*o*NPOE), ferrocenemethanol were obtained from Sigma-Aldrich. High molecular weight poly(vinyl chloride) (PVC) was from Merck (Darmstadt, Germany). Volatile solvents extra pure cyclohexanone (CH) and HPLC grade tetrahydrofuran (THF) were obtained from Vekton (St. Petersburg, Russia). Inorganic salts analytical grade were from Reaktiv (Moscow, Russia). All aqueous solutions were prepared with deionized water with resistivity 18.2 MOhm·cm (Milli-Q Reference, Millipore, France).

The membrane cocktails were prepared by dissolving appropriate amounts of ionophore, ion exchanger, background electrolyte and PVC in THF. The dry mass of the cocktail was 15% for K- and Li-ISEs and 20% for Ca-ISE. The cocktail were stirred until the components were completely dissolved using a roller-mixer Selecta Movil Rod (Barcelona, Spain). For the membrane compositions see Table 1.

The ISEs of classical construct with an internal solution and an internal reference electrode were used in the study. ISEs selective to Li^+^- and Ca^2+^-ions were prepared as follows. To obtain membranes the cocktail were cast on glass Petri dishes. The dishes were closed with filter paper to slow down the evaporation of THF. After the complete evaporation of THF (overnight) master membranes with a thickness of 90–110 µm were obtained. The thickness of these membranes was evaluated with a caliper, from the difference in the thickness of two glasses with a membrane sandwiched between them and the thickness of the same two glasses without membrane. Disks with a diameter of 10 mm were cut from the master membrane and glued to PVC bodies with an outer diameter of 12 mm and an inner diameter of 8 mm. A solution of PVC in CH was used as the glue.

**Figure 1:**
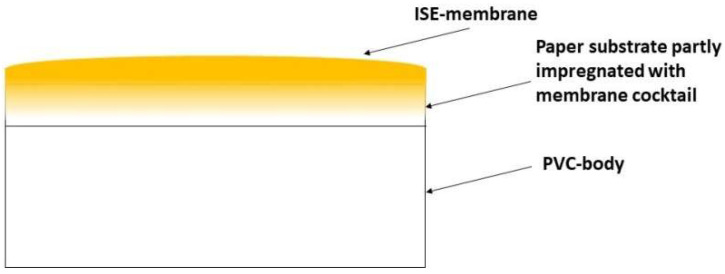
Side-view of the ISE with a membrane formed on the paper substrate.

K-ISEs were prepared in another way. Paper discs (80 g/m^2^) with diameter of 10 mm were glued to PVC-bodies and then 160 µL of the cocktail was drop cast on this paper substrate. The estimated thickness of the membrane was 300 µm. The thickness of the K-selective membrane was evaluated by calculations based on the area of the paper substrate and the volume of the applied cocktail with a known dry weight. However, this value may overestimate the membrane thickness since a part of the cocktail impregnates the substrate, as is shown in Figure 1, so only the upper part of the membrane comprises a continuous layer.

Initially, K-, Li- and Ca-ISEs were filled with 0.01 M solutions of the respective chlorides and conditioned in the same solutions for at least 1 day. After that, the potentiometric calibration was held to ensure that the electrodes operate correctly. Zero current potentiometric measurements were performed with EMF 16 multichannel potentiometric interface (Lawson Labs, Inc., Malvern, PA, USA) equipped with a PC for data acquisition. The reference electrode was a single junction Ag/AgCl electrode in saturated KCl, with a salt bridge with a limited leak of KCl. A chlorinated silver wire was used as the internal electrode in the ISEs. Calibrations were carried out from 10^−1^ M down to 10^−8^ M respective solution: KCl, LiCl, CaCl_2_, using automatic burette Metrohm 700 Dosino controlled by a Metrohm 711 Liquino Controller (Metrohm, Buchs, Switzerland).

Non-zero current measurements were held with Potentiostat-Galvanostat Autolab 302N with a frequency response analyzer module FRA 2 (Metrohm, Utrecht, The Netherlands). Electrochemical impedance spectra were recorded in potentiostatic mode with the excitation magnitude of ±5 mV around the open circuit potential (OCP) within a frequency range from 100 kHz to 0.1 Hz or 0.01 Hz.

**Figure 2:**
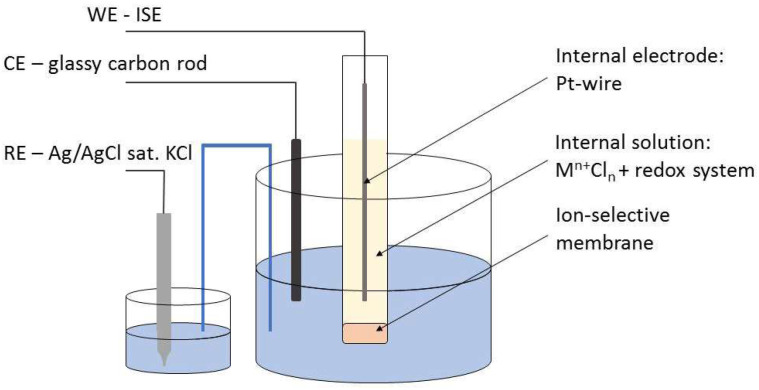
The cell used in voltammetric mode.

Cyclic voltammograms (CVs) were recorded using a scan rate of 100 mV/s (unless otherwise stated). During CV measurements, a platinum electrode (EPL-02, Izmeritel, Gomel, Belarus) was used as an internal electrode, and the inner filling solution contained not only respective chlorides (0.01 M) but also a redox system required to produce redox peaks: K_3_Fe(CN)_6_/K_4_Fe(CN)_6_ (2 × 10^−4^ M/2 × 10^−4^ M) for K-ISEs and ferrocenemethanol (1 × 10^−3^ M) for Li- and Ca-ISEs. The cell construct used in voltammetric mode is shown in Figure 2.

## 4. Results and Discussion

### 4.1. Obtaining Thin Membranes for Classical ISEs

It was suggested that to observe relatively high currents in the voltammetric mode, it is necessary to work with low-resistant objects, which means the necessity to reduce the resistance of ion-selective membranes. This can be achieved in different ways, including varying their geometric parameters. To obtain fairly thin membranes, which must also be durable enough to be used in classical construction, different techniques have been utilized. A new method of membrane formation was suggested: to glue paper substrate to the top of the electrode body and then drop-cast a membrane with a rather small thickness. This approach was utilized in preparation of K-ISEs. However, the selectivity of membranes obtained in this way was low until the estimated thickness (calculated from surface area and cocktail volume) reached 300 µm. This could be due to insufficient paper smoothness and protruding paper fibers, which may induce leaks and therefore negatively affect selectivity. Since the technology of gluing disks with even smaller thicknesses right to the top of the PVC-bodies (without any substrate) was worked out, thin Li- and Ca-selective membranes with sufficient selectivity were obtained by this method. The thickness and the resistance of the obtained membranes are shown in Table 2. Although K-selective membrane is ca. 3 times thicker than that of Li-, its resistance is almost 3 times lower, apparently due to the difference in the plasticizers: *o*NPOE in K-selective membrane and DOS in Li-selective membrane and also due to the presence of background electrolyte in K-selective membrane.

### 4.2. Potentiometric Study of Electrodes Performance

Before using the obtained ISEs in voltammetric mode, their potentiometric response and selectivity were checked. Initially, the potentiometric calibration was held with the respective chloride solutions as inner filling. Sodium ions (as NaCl) were used as suitable interference to check the selectivity. As shown below (see Section 4.3.2), voltammetric measurements with Li-ISEs required a background electrolyte and MgCl_2_ was chosen as suitable candidate. Therefore, in the case of Li-ISEs, Mg^2+^ interference was also studied. The selectivity was measured by the separate solutions method with 0.1 M NaCl or 0.1 M MgCl_2_.

**Figure 3:**
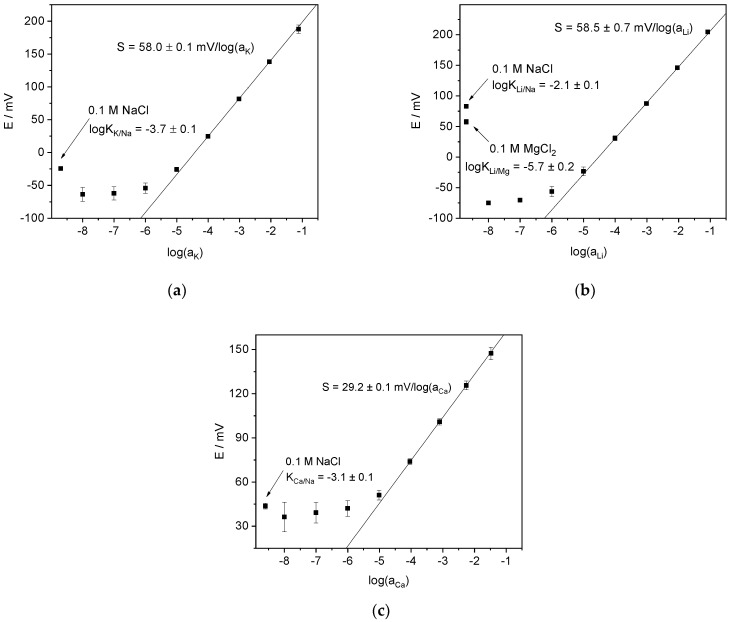
Potentiometric calibration plots of the ISEs: (**a**) K-ISEs, (**b**) Li-ISEs, (**c**) Ca-ISEs.

The results: calibration plots, slope values and selectivity coefficients are shown in Figure 3. The selectivity coefficients of K-ISE (and Li-ISE) over Na^+^ were calculated by the Nikolsky equation:

(6) $$E=E^{0}+S_{K}log\left(a_{K}+Ka_{Na}\right)$$

In the case of differently charged ions, e.g., Ca^2+^ over Na^+^ or Li^+^ over Mg^2+^, more complicated equations were derived [48,49]

(7) $$E=E^{0}+2S_{Ca}log\left({\left(a_{Ca}+\frac{1}{4}Ka_{Na}^{2}\right)}^{1/2}+{\left(\frac{1}{4}Ka_{Na}^{2}\right)}^{1/2}\right)$$

(8) $$E=E^{0}+S_{Li}log\left(\frac{1}{2}a_{Li}+\frac{1}{2}{\left(a_{Li}^{2}+4Ka_{Mg}\right)}^{1/2}\right)$$

However, in our case (separate solutions) Equation (7) was reduced to the equation recommended by IUPAC [50]

(9) $$E=E^{0}+S_{Ca}log\left(a_{Ca}+Ka_{Na}^{2}\right)$$

which was used for calculations.

The values of the selectivity coefficients are consistent with those reported elsewhere [51]. When ions are of the same charge, such as K^+^, Li^+^ and Na^+^, the selectivity coefficient K describes the interference in an intuitively clear way: $K^{-1}$ roughly shows the tolerable excess of the interfering ion. This is not so simple if ion charges are different. Due to the difference in the values of the Nernst factor, the selectivity coefficient of Ca-ISE over Na^+^ apparently overestimates the interference from Na^+^ ions. On the contrary, in the case of Li^+^, the interference from Mg^2+^ is stronger than one could expect from the selectivity coefficient.

**Figure 4:**
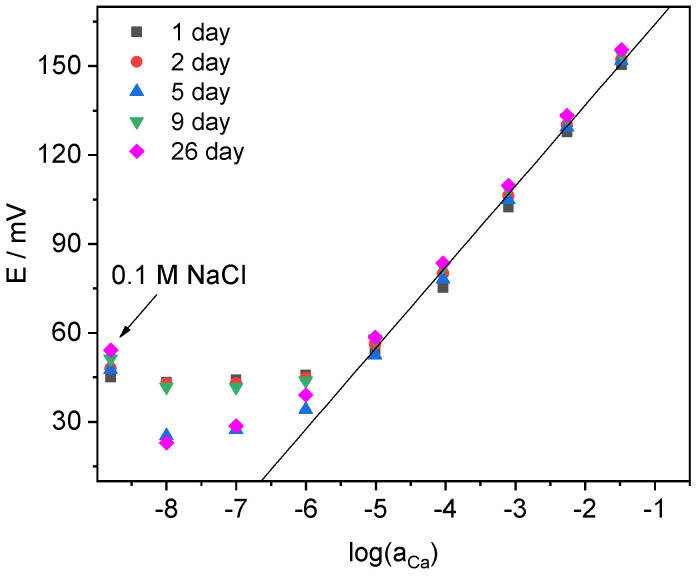
Potentiometric calibration plots of Ca-ISE filled with mixed solution 0.01 M CaCl_2_ + 0.001 M ferrocenemethanol recorded at different days.

Since the idea of the application of classically designed ISEs in voltammetry relies on the usage of a redox system as a component of the internal solution, the impact of ferrocenemethanol on the electrode response has been studied. Ca-ISEs were filled with the solution, containing CaCl_2_ (1 × 10^−2^ M) and ferrocenemethanol (1 × 10^−3^ M) and used for 26 days without the replacement of this internal solution. As shown in Figure 4, ferrocenemethanol does not affect the electrode function and selectivity, at least, within 26 days of contact with the membrane. Electrodes’ parameters averaged over time are as follows: E^0^ = 191.4 ± 2.7 mV, slope (S) = 27.3 ± 0.4 mV/log(a_Ca_) and logK_Ca/Na_ = −3.1 ± 0.1. Thus, for electrodes containing ferrocenemethanol in internal solution parameters observed in zero current mode demonstrate a sufficient reproducibility.

### 4.3. Voltammetric Measurements with ISEs Containing Internal Solution

#### 4.3.1. Ca-ISE

**Figure 5:**
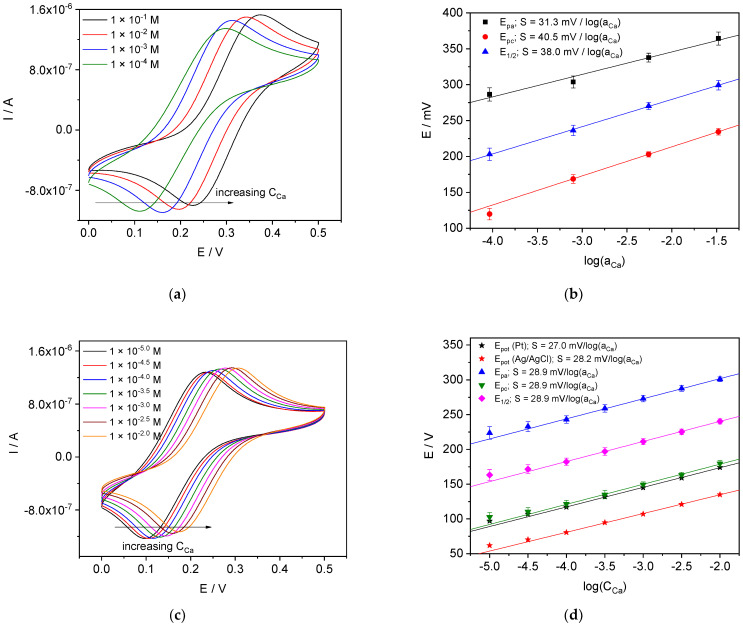
Results obtained with Ca-ISE. (**a**) CVs in pure CaCl_2_ solutions; (**b**) peak potentials in pure CaCl_2_ vs. log(a_Ca_); (**c**) CVs recorded in CaCl_2_ solutions with 0.1 M NaCl background; (**d**) peak potentials in CaCl_2_ with 0.1 M NaCl background vs. log(C_Ca_).

The CVs recorded with Ca-ISEs in pure CaCl_2_ solutions and in CaCl_2_ solutions containing 0.1 M NaCl as a background electrolyte are shown in Figure 5. The results are consistent with those expected from theoretical considerations: peak potentials show Nernstian behavior, while peak currents are practically the same regardless the concentration of CaCl_2_, analogous to reported for SC-ISEs [37,39,40,41,42]. Apparently, the deviations of the slopes from the ideal value of 28.1 mV/log(a_Ca_), in the case of pure solutions of CaCl_2_, are due to the impact from the non-constancy of the resistance of the ISE membrane [49,50,51,52] as well as of the solution. In our recent works [52,53,54,55,56], we demonstrated that membrane resistance increases along with the dilution of the solution. We suggested a hypothesis relating the change of the resistance to water uptake by the ISE membranes [56,57]. Furthermore, the resistance depends on the ionic strength rather than on the concentration of the individual electrolyte [57].

We therefore performed voltammetric measurements using CaCl_2_ solutions with NaCl background. The selectivity of Ca-ISEs was sufficient to operate with a background electrolyte concentration of almost four orders of magnitude higher than the analyte concentration (Ca^2+^). The use of background electrolyte ensures constant resistance of the membrane and solution and allowed better results to be obtained. In case of the measurements with 0.1 M NaCl the observed slopes of the peak potentials vs. log(C_Ca_) are closer to Nernstian, see Figure 5b,d. *E_pa_* refers to the oxidation peak, *E_pc_* to the reduction peak and *E*_1/2_ corresponds to the mean of anodic and cathodic peak potentials: $E_{1/2}=\frac{E_{pa}+E_{pc}}{2}$. These potential peak shifts almost coincide with those obtained in potentiometric mode (*E_pot_*, Figure 5d): the dependences are almost parallel to each other, either with the platinum internal electrode (black stars in Figure 5d) or silver chloride internal electrode (red stars in Figure 5d). The deviation from the linearity observed at low concentrations is the same for all of five sets of data. This proves that the origin of the potential shifts observed in voltammetry is the same as for the potentiometric potential shifts and corresponds to the potential change at the interface between the membrane and the external solution.

#### 4.3.2. Li-ISE

**Figure 6:**
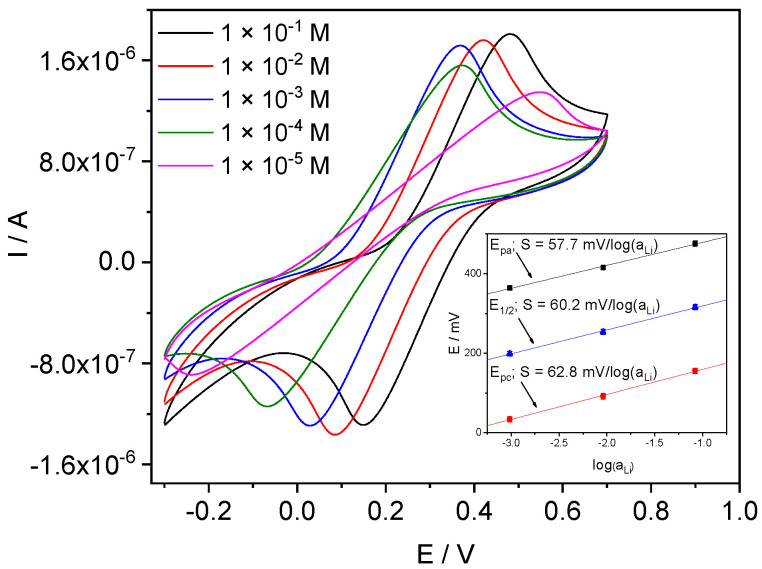
CVs with Li-ISE obtained in pure solutions of LiCl, scan rate 100 mV/s. Inset: peak potentials in pure LiCl vs. log(a_Li_) from 10^−3^ M to 10^−1^ M.

**Figure 7:**
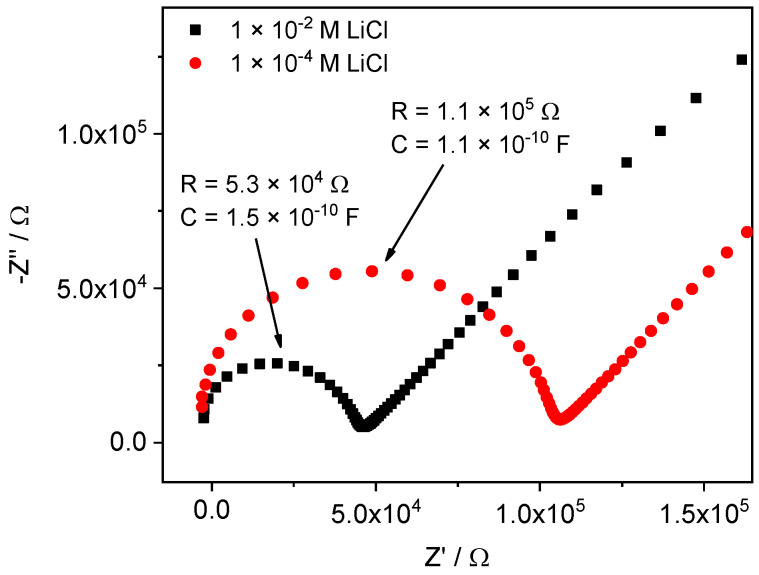
Impedance spectra of Li-ISE obtained in pure 10^−2^ M LiCl and 10^−4^ M LiCl.

The CVs recorded with Li-ISEs in pure solutions are shown in Figure 6. In the range of 10^−1^ M–10^−3^ M LiCl the peak potential shifts with a Nernstian slope (inset). The peak current remains practically the same and the voltammograms are identical in shape. However, the CV curves become significantly distorted while measuring in pure LiCl solutions with concentrations lower than 10^−4^ M. In principle, this may be due to the increase of the resistance of the solution, or of the membrane, or both. However, in this case the impact from the membrane is predominating, as indicated by impedance spectra. Indeed, the spectra recorded in the 10^−2^ M LiCl and 10^−4^ M LiCl show almost twofold resistance increase, see Figure 7. We assume that the change of the curves shape obtained in 10^−4^ M LiCl and below is a consequence of the increase in the membrane resistance.

**Figure 8:**
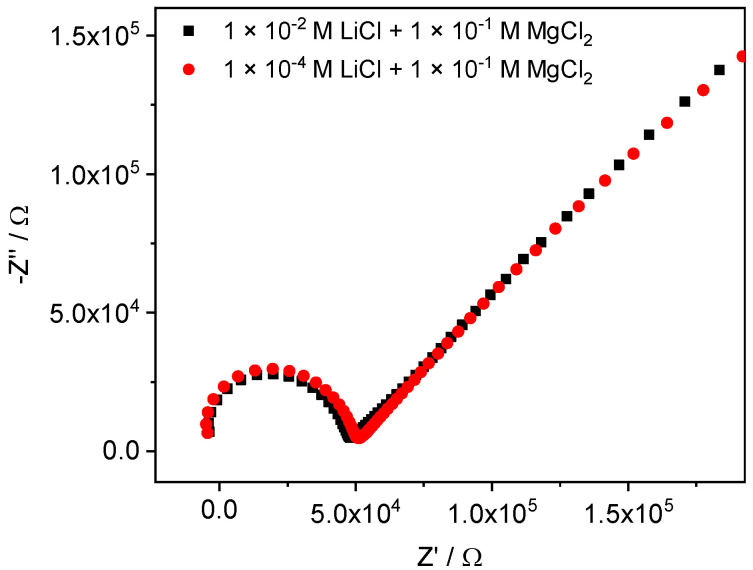
The impedance spectra of Li-ISE obtained in 10^−2^ M LiCl and 10^−4^ M LiCl solutions containing 0.1 M MgCl_2_ as a background.

**Figure 9:**
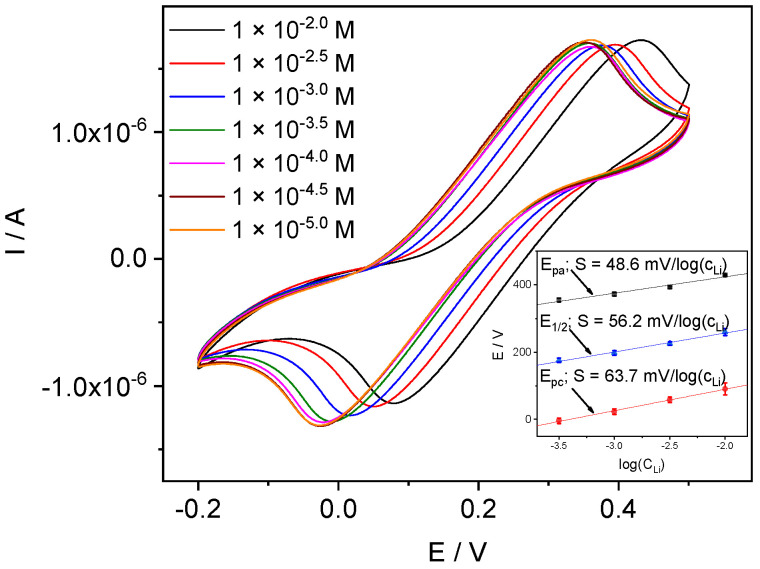
CVs with Li-ISE obtained in LiCl solutions containing 0.1 M MgCl_2_. Inset: peak potentials vs. log (a_Li_) from 10^−3.5^ M to 10^−2^ M.

To fix the ionic strength and, as follows from our assumptions, also the membrane resistance, further measurements were made using the solutions of LiCl containing 0.1 MgCl_2_ background. Indeed, in the presence of 0.1 M MgCl_2_, the membrane resistance in 10^−2^ M LiCl and 10^−4^ M LiCl became almost the same, see Figure 8. Voltammograms recorded with a 0.1 M MgCl_2_ background (see Figure 9) do not demonstrate such distortions, as in the case of dilute solutions without background. However, peak potentials followed Nernstian law only down to 10^−3.5^ M LiCl, apparently, due to insufficient selectivity in the presence of 0.1 M MgCl_2_.

**Figure 10:**
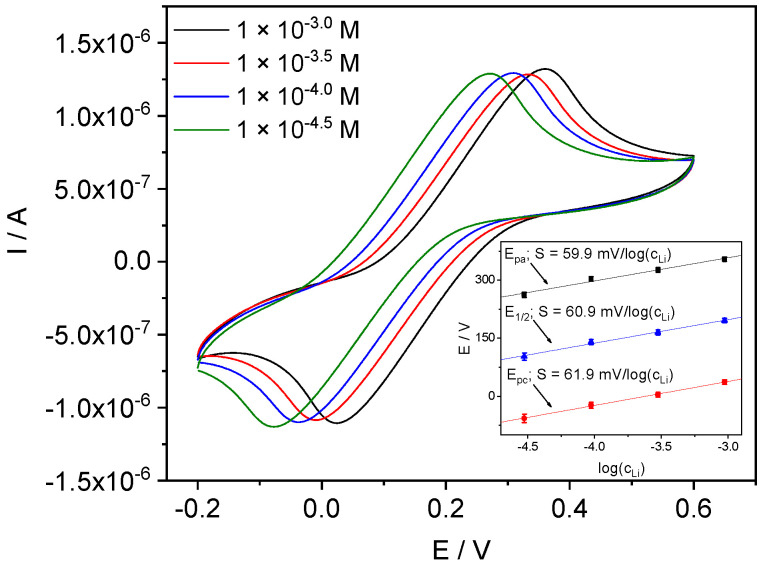
CVs with Li-ISE obtained in LiCl solutions with 0.001 M MgCl_2_. Inset: peak potentials vs. log(a_Li_) from 10^−4.5^ M to 10^−3^ M.

To achieve a better detection limit of Li^+^, voltammetric measurements were held with the 0.001 M MgCl_2_ background. The results presented in Figure 10 demonstrate that in this case the linear range of the potential shift with the slope close to Nernstian continues to lower concentrations: logC_Li_ = −4.5 instead of −3.5 when the 0.1 M MgCl_2_ background was used.

#### 4.3.3. K-ISE

**Figure 11:**
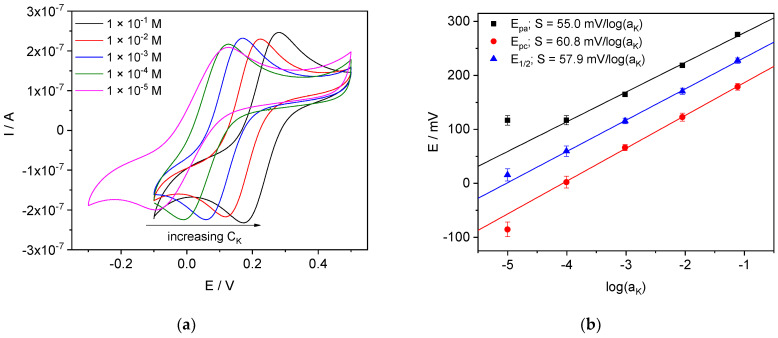
Results obtained with K-ISE. (**a**) CVs in pure KCl solutions; (**b**) peak potentials vs. log(a_K_).

CVs recorded with K-ISE in pure KCl solutions are shown in Figure 11. Well-developed voltammograms were obtained at KCl concentration down to 1 × 10^−4^ M even without background electrolyte. Only at the lowest concentration: 1 × 10^−5^ M, the CV is somewhat distorted, suggesting increasing the resistance of the membrane in contact with this solution. As in the case of Ca- and Li-ISEs, we ascribe the increase of the membrane resistance to water uptake, which increases along with the dilution of the aqueous solution. However, the effect is less pronounced than with Li-ISE, which showed distorted CVs at 10^−3.5^ M LiCl already. We believe that the reason for this difference in the performance of potassium and lithium ISEs is due to the difference in their thickness. It is known that water in membranes is distributed non-uniformly, even after long contact with solutions: water is located primarily in the vicinity of the membrane/solution interface [56,58,59,60,61,62,63,64]. Therefore, the thinner the membrane is, the larger the part enriched in water. Respectively, the resistance of thicker membranes is less sensitive to the ionic strength of the solution. Hence, the effect of the resistance non-constancy for thicker K-selective membranes (ca. 300 µm) is observed at lower ionic strength, than those observed for thinner Li-ISEs (ca. 100 µm).

#### 4.3.4. Scan Rate Dependence

**Figure 12:**
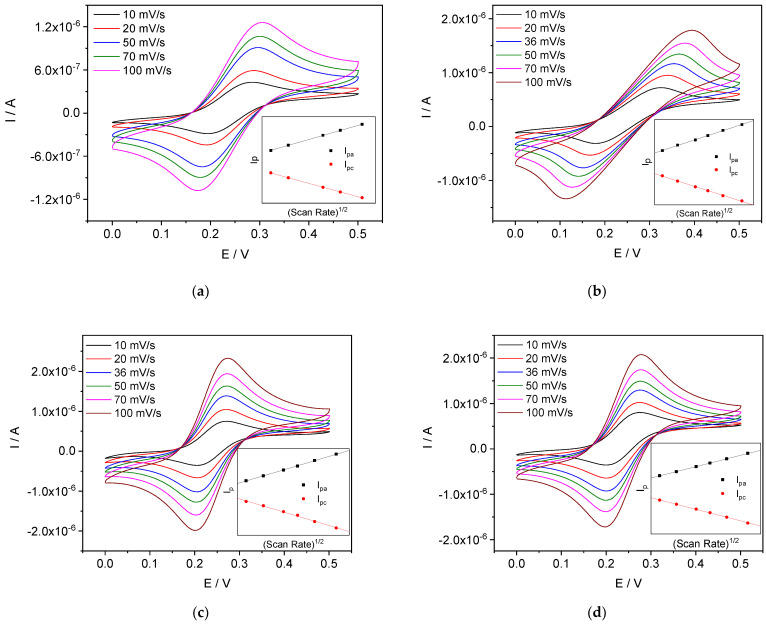
CVs obtained at different scan rates for (**a**) Ca-ISEs in 0.01 M CaCl_2_; (**b**) Li-ISEs in 0.01 M LiCl; (**c**) platinum wire in a solution containing 1 × 10^−3^ M FcMeOH and 1 × 10^−2^ M CaCl_2_; (**d**) platinum wire in a solution containing 1 × 10^−3^ M FcMeOH and 1 × 10^−2^ M LiCl. Insets: peak currents vs. square root of scan rate.

Cyclic voltammograms obtained with Ca^2+^- and Li^+^-ISEs in 0.01 M CaCl_2_ and 0.01 M LiCl, respectively, at different scan rates are shown in Figure 12a,b. Both ISEs show the linearity of the peak currents vs. the square root of scan rate (insets). This is in contrast with the results obtained by Bakker’s group for thin membranes (200–300 nm) [34]. The linear dependence of current upon square root of scan rate indicates that the process is controlled by diffusion. The peak potentials are nearly independent of scan rate for Ca-ISEs and more dependent in the case of Li-ISEs. This difference may be due to the higher resistance of Li-ISEs.

For comparison, CVs of platinum wire in the respective internal solutions, without membrane, are shown in Figure 12c,d. The results are consistent with literature data on the electrochemical behavior of FcMeOH/Fc^+^MeOH on platinum [65], gold [66] and glassy carbon [67]: the redox process FcMeOH/Fc^+^MeOH on such electrodes is diffusion limited. Obviously, the difference between our results and those reported by Bakker’s group is due to the different nature of the redox-active system in the electrodes: thin film of a CP (Bakker, reaction limited) and FcMeOH/Fc^+^MeOH in a bulky aqueous solution (this work).

## 5. Conclusions

This is the first report on the voltammetric counterpart of classical ISEs with internal aqueous solution, which are normally used in zero-current mode. The voltammetric response in this case originates from redox processes at the interface between the platinum wire (internal electrode) and redox system: ferrocenemethanol or Fe^3+^/Fe^2+^ (in form of ferrocyanides) in the internal solution. For such ISEs, it is shown theoretically and confirmed experimentally that the dependence of oxidation and reduction peak potentials on the sample composition obeys the Nernst law. This dependence originates from ion exchange processes at the interface between the ISE membrane and the external solution (sample). On the other hand, the peak currents almost do not depend on the sample composition. This is in contrast with classical voltammetry and consistent with data reported by Bakker’s group for SC-ISEs with ultra-thin membranes (200–300 nm) [37,40,41]. Unfortunately, the extremely small thickness of the latter membranes results in an extremely short lifetime, which significantly limits their practical application. Furthermore, the re-coating of the thin film on the glassy carbon substrate of the electrode was carried out every time if the solution needed to be replaced [44].

The key novelty of this work is overcoming this problem through the use of ISEs of classical design with relatively thick membranes (100–300 µm), so that the lifetime of the ISEs is about one month. This makes voltammetry with ISEs promising for practical applications. Importantly, the presence of such redox systems as ferrocenemethanol or ferrocyanides in internal solution does not hamper either sensitivity or selectivity of ISEs, which allows their use in complete analogy with conducting polymers in solid-contact ISEs.

The detection limits in voltammetric measurements with ISEs are in 1–1.5 orders of magnitude worse than in zero-current potentiometry [39]. We ascribe this to the increase of the membrane resistance in diluted solutions. In this respect, it is important that the use of a suitable background electrolyte allows for better detection limits in voltammetric measurements with ISEs as compared with measurements in pure solutions. Since practically relevant tasks assume samples with complicated matrixes, this finding is of special importance.

Our further studies will be devoted to voltammetry with membranes containing several ionophores to be used in multisensory arrays.

## Figures and Tables

**Table 1:** Membrane compositions (weight percent and concentration (Mol/kg of plasticizer) of the components).

Ion	Neutral Ionophore	Ion Exchanger	Plasticizer	PVC	Background Electrolyte
K^+^	Valinomycin 5.4%, 0.08 m	KClTPB 1.2%, 0.04 m	*o*NPOE 60.4%	30.2%	ETH 500 2.8%, 0.04 m
Li^+^	Li-ionophore VIII 2.6%, 0.05 m	NaTFPB 1.4%, 0.025 m	DOS 64.0%	32.0%	-
Ca^2+^	ETH 1001 0.9%, 0.02 m	NaTFPB 0.6%, 0.01 m	*o*NPOE 64.7%	32.3%	ETH 500 1.5%, 0.02 m

**Table 2:** Thickness and resistance of the obtained ISEs.

Ion	Thickness, µm	Resistance, Ω
K^+^	300 ± 30	(1.28 ± 0.10) × 10^4^
Li^+^	90 ± 10	(3.21 ± 0.86) × 10^4^
Ca^2+^	110 ± 10	(1.26 ± 0.17) × 10^4^

## Data Availability

The data presented in this study are available in this article.
